# A policy in stagnation: addressing inequalities in the distribution of emergency obstetric and neonatal care in Ghana

**DOI:** 10.3389/fgwh.2025.1614200

**Published:** 2025-07-18

**Authors:** Ephraim Senkyire, Gloria Senkyire, Rullmann Twi Owusu, Ernestina Asiedua

**Affiliations:** ^1^ Department of Nursing and Midwifery, Ga West Municipal Hospital-Ghana Health Service, Accra, Ghana; ^2^ Department of Accountancy, Faculty of Business and Management Studies, Sunyani Technical University, Sunyani, Ghana; ^3^ Department of Marketing and Supply Chain Management, School of Business, University of Cape Coast, Cape Coast, Ghana; ^4^ Department of Maternal and Child Health, School of Nursing and Midwifery, University of Ghana, Accra, Ghana

**Keywords:** EmONC, inequalities, maternal mortality, distribution, policy, Ghana, emergency obstetric and neonatal care

## Abstract

Maternal health has been a focal point of global attention since the 1980s, with initiatives like Safe Motherhood, Millennium Development and Sustainable Development Goals aiming to improve the well-being of women and infants worldwide. Despite these efforts, high maternal and neonatal mortality rates persist, particularly in middle-income countries, including Ghana, highlighting the need for urgent action. From 2000 to 2020, Ghana successfully halved its maternal mortality ratio from 499 to 263 deaths per 100,000 live births through various interventions and strategies, which is still higher than the global average. Emergency obstetric and neonatal care (EmONC) plays a vital role in preventing maternal and neonatal deaths, yet disparities in its distribution and delivery exist, particularly in Ghana. An evaluation of EmONC facilities in Ghana highlighted the challenges of infrastructure, human resources, logistics, and equipment in meeting the World Health Organisation standards for EmONC within national, regional, rural, and urban health facilities. This critical analysis paper aims to highlight these challenges and propose comprehensive solutions for improved delivery of EmONC services. Addressing these challenges requires comprehensive efforts to improve infrastructure, human resources, and supply chain logistic support. A two-pronged approach is recommended. One recommendation focuses on upgrading existing facilities and recruiting and retaining healthcare professionals in rural and underserved areas. The second recommendation calls for increasing the capability of delivery of EmONC by improving training efficiency and focusing on facilities missing only one or two of the seven key services required for basic emergency obstetric and newborn care.

## 1 Introduction

Maternal mortality refers to deaths occurring in women during pregnancy or within 42 days after childbirth (1). In contrast, pregnancy-related death encompasses any death that occurs during pregnancy or within one year after its termination, regardless of the cause, including accidental or incidental factors (2). The maternal mortality ratio (MMR), an estimate of maternal mortality per 100,000 live births within a specific time, is a metric for evaluating obstetric care quality and overall healthcare delivery (3). Globally, women experience complications during pregnancy or postpartum, resulting in 223 deaths per 100,000 live births annually (4, 5). Although recent efforts have decreased MMRs in low-middle-income countries, high rates persist (3, 6–9). Moreover, 2.3 million neonates die annually, and 1.9 million are stillborn globally (10, 11). Additionally, numerous women and infants suffer from disabilities related to childbirth. Most of these mortalities and disabilities are preventable. Although some complications during pregnancy and birthing cannot be foreseen or averted, almost all of them can be effectively managed with competent and efficient emergency obstetrics and newborn care (EmONC) (4).

From 2,000 to 2020, Ghana has successfully halved its maternal mortality ratio (MMR) from 499 to 263 deaths per 100,000 live births, but this rate is still higher than the global average (12). Various policy interventions by the government have contributed to this success (3). These initiatives include the National Health Insurance (NHI) Act 2003, which aims to provide all residents with quality, affordable healthcare services (3). The free maternal healthcare policy, Safe Motherhood Programme, Life Saving Skills Programme, Integrated Management of Childhood Illness, Focused Antenatal Care, and Accelerated Child Survival and Development have also contributed to reducing maternal and neonatal mortality (13, 14). The Ghana Essential Health Intervention Programme (GEHIP) has bolstered the Community Health Planning Services (CHPS), which ensures healthcare accessibility in rural communities (14). The CHPS programme is a national strategy aimed at enhancing healthcare delivery within communities and strengthening primary healthcare in rural areas. This initiative was established to offer a range of services, including health education, health promotion, management of minor ailments, community mobilisation for health initiatives, referral services, and home visits (15). Despite these concerted efforts, maternal and newborn deaths remain elevated (16) compared to the global standard (5, 17).

EmONC encompasses a set of interventions for mothers and newborns experiencing severe complications in the course of pregnancy, delivery, and postpartum (18). It is estimated that accessible and high-quality EmONC could prevent three-fourths of maternal mortality (19). However, its effectiveness relies on the readiness of health facilities to deliver care, the quality of care provided, and the functionality, timeliness, and organisation of referral systems within a given context (18).

High quality EmONC entails enhancing the capabilities of health facilities to deliver essential services aimed at preventing avoidable deaths during childbirth (20). It involves upgrading selected health centres and referral hospitals to ensure an adequate reserve of basic drugs, materials, and equipment and trained healthcare professionals to deliver quality services consistently (20). EmONC is categorised into basic EmONC (BEmONC) and comprehensive EmONC (CEmONC). BEmONC facilities provide essential functions such as administering oxytocic drugs and antibiotics as well as performing procedures like manual removal of the placenta and basic neonatal resuscitation. CEmONC facilities provide all BEmONC functions along with additional services such as caesarean sections and blood transfusions (20). The UN guidelines recommend a minimum of five EmONC facilities per 500,000 people, with not less than one offering comprehensive care (21). In contrast, Ghana has set a more stringent target, aiming for at least five EmONC facilities per 200,000 people, including one offering comprehensive care (19).

Stakeholder perspectives on substandard EmONC in Ghana underscore several key issues. These include the concentration of EmONC services in central locations, inadequate funding, limited opportunities for hands-on training, delays in the recruitment of newly trained essential personnel, and a lack of provider engagement in the profession (22). The “three delays’ model aids in pinpointing potential points of delay in handling obstetric complications (23). The first, second and third delay encompasses delays in seeking medical attention, locating and accessing the healthcare facility and receiving care at healthcare facilities, which can stem from various factors such as facility organisation, care quality, and the availability of staff and equipment (24). All hospitals in Ghana are expected to provide the full range of EmONC services (25). However, an evaluation of EmONC facilities in Ghana highlighted infrastructure, human resources, logistics and equipment challenges in meeting the World Health Organisation standards for EmONC within all health facilities (19, 25). Addressing these challenges is crucial to efficiently managing obstetric emergencies (23). Here, we explore the context of these challenges and propose comprehensive solutions.

## 2 Infrastructure

Ghana's health system is characterised by a high degree of decentralisation. It is managed by the Ministry of Health (MoH) in collaboration with sub-agencies such as the Ghana Health Services (GHS) and the National Health Insurance Authority (NHIA). The MoH is primarily responsible for policy and regulatory oversight and managing teaching hospitals. At the same time, the GHS is tasked with promoting access to health services at the community, sub-district, district, and regional levels. The NHIA administers the National Health Insurance Scheme (NHIS) (26). Both the public and private sectors are vital stakeholders in the healthcare system. The public sector is structured into several tiers: national (teaching hospitals), regional hospitals, district (public and mission hospitals), sub-district (public health centres), maternity homes and community (CHPS). Modern healthcare services are supplemented by traditional medicine, which remains popular, especially among rural populations (27).

In 2011, Ghana undertook a national survey of its 1,268 healthcare facilities. Among these were 273 District hospitals, three teaching hospitals, 518 health centres, nine regional hospitals, 139 CHPS, 165 Maternity homes, and 161 health clinics (19). Facilities were categorised as BEmONC if they offered all seven BEmONC signal functions (28) such as administering oxytocic drugs, antibiotics, and anticonvulsants, manual removal of the placenta and basic neonatal resuscitation within the preceding three months, while those providing all the seven BEmONC plus caesarean sections and blood transfusions were graded as CEmONC (19). Using the more stringent requirement of a minimum of five EmONC facilities per 200,000 individuals, including at least one facility offering CEmONC, there should ideally be 485 basic facilities and 121 comprehensive facilities. However, the national assessment found that only 13 facilities met the criteria for BEmONC, resulting in a shortfall of 472 facilities. Additionally, 76 comprehensive facilities meet the standard for CEmONC, leaving a gap of 45 (19). Conversely, according to the United Nation Development programme, a 2020 assessment revealed that only 52 health facilities had the capacity to deliver CEmONC, falling significantly short of the national target of 155 facilities (29).

From 2022 to 2023 Ghana Harmonised Facilities Assessment (GHFA) report, of the 1,421 health facilities surveyed, 66% of facilities reported offering BEmONC. Only 15% delivered all seven BEmONC signal functions. 39% of polyclinics and 10% of health centres offer all seven BEmONC signal functions. However, 17% of district hospitals and 49% of general hospitals do not offer all seven basic EmONC (BEmONC) signal functions (25). On the other hand, the availability of CEmONC services remains limited. Only 16% of facilities reported offering caesarean section services, and 18% provided blood transfusions. While 20% of facilities identified as offering CEmONC services, just 10% met the full criteria by providing all nine signal functions. Although 15% of health centres claimed to offer CEmONC, a closer look revealed that fewer than 2% actually provided either blood transfusion or caesarean section services, indicating a significant gap between reported and actual capacity. Yet, 17% of district hospitals and 53% of general hospitals lack the full complement of nine comprehensive EmONC (CEmONC) signal functions (25).

**Figure 1:**
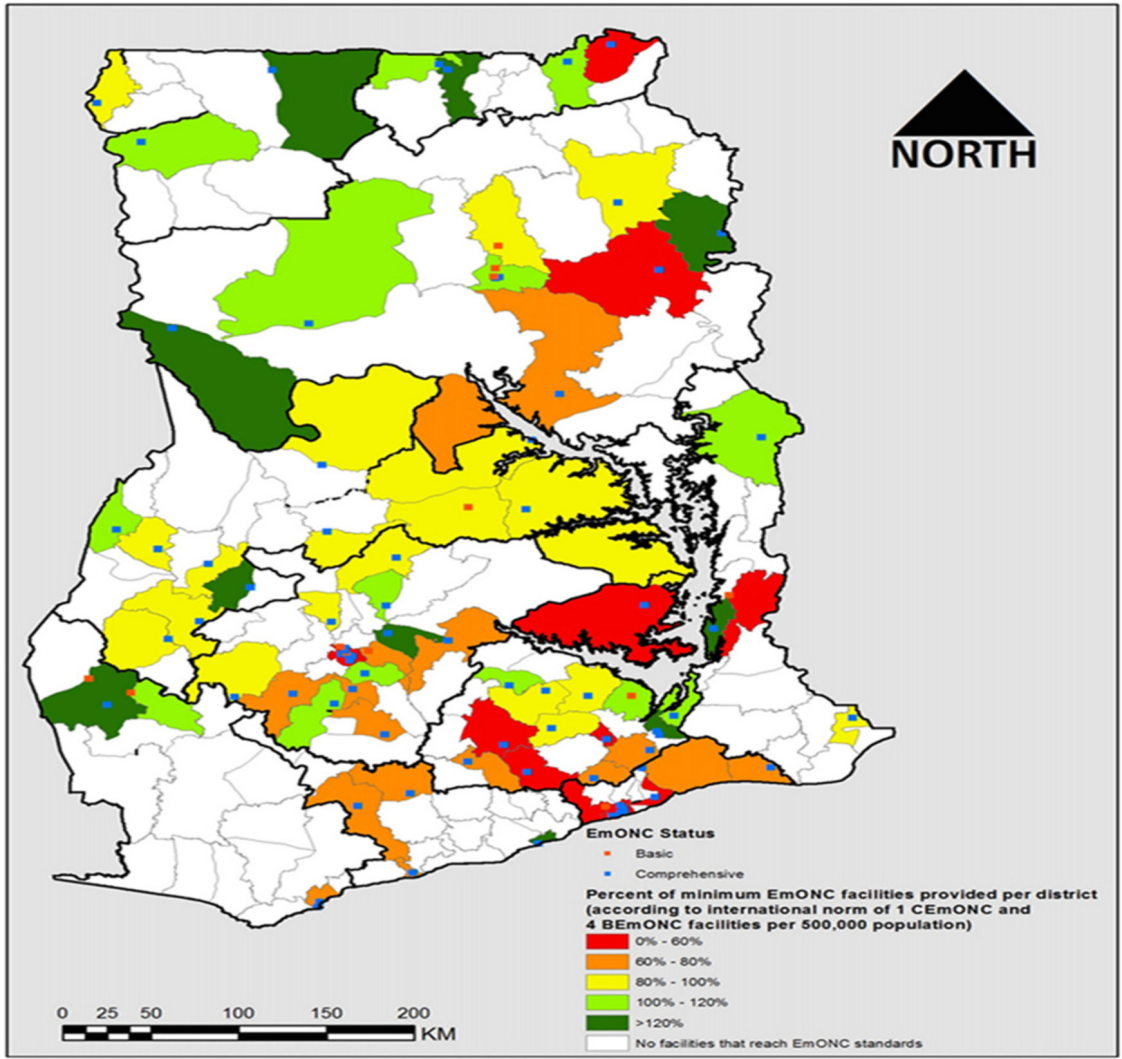
The percentage of the minimum acceptable number of fully functioning EmONC facilities per district (30).

Bosomprah et al. (2016) conducted a study aimed at policy recommendations for addressing deficiencies in the delivery of signal function services and their distribution throughout the country (see Figure 1). Results found that out of 1,159 maternity facilities surveyed, only 89 (7.7%) were equipped to provide all the necessary basic or comprehensive EmONC signal functions once in the three months preceding the national survey. Accordingly, just 21% of births in healthcare facilities occurred in operational EmONC facilities, while a further 30% took place in facilities lacking one or two basic signal functions. Thus, addressing these deficiencies in signal functions could potentially improve the ratio of births in fully operational facilities to over 50% (30). Regionally, there is significant variation in the availability of EmONC facilities, with most facilities located in urban areas, and a few located in rural areas especially in the three northern regions (upper west, upper East and Northern) as CHPS and health centres, which is not mandated or cannot provide EmONC (19, 25, 31). The assessment (19) included 216 facilities from the Greater Accra region, 148 facilities from the Ashanti region, and 96 facilities from the Upper West region. The Ashanti and Greater Accra Regions have the largest gaps, with 97 and 87 facilities, respectively, while the Upper West Region has the smallest gap, with only 12 facilities that meet facility per population. Also, 80% of health facilities in the northern region of Ghana failed to meet the criteria for providing EmONC (31). For CEmONC facilities, the Greater Accra Region faces the largest shortfall of 13 facilities per population. Regarding Basic EmONC, Ashanti has the greatest deficit, needing 91 more facilities, whereas Upper West has the smallest deficit, needing only 14 facilities in addition to existing facilities in order to meet expected access points per population of individual region (19). Per recent GHFA report, nearly all regions indicated that at least 50% of their sampled facilities provided some BEmONC services (25). With the exception of the Greater Accra region, all other regions had fewer than 30% of facilities capable of delivering CEmONC services (25).

The lack of medications and personnel capable of dealing with pregnancy complications at the appropriate level of a facility affects referral rates and increases delays in care. Bailey et al. (32) identified a strong correlation between readiness to perform EmONC and referral to the next level of care. In this study, over half of births took place in district hospitals but only 21% of these were prepared to treat postpartum haemorrhage and abortion complications. As expected, facilities with adequate supplies numbers of midwives, and enhanced communication and transportation systems referred out fewer women.

An additional infrastructure problem is the unequal geographical distribution of prepared health facilities, which can result in poor maternal and neonatal outcomes. In contrast, a study of four districts in Ghana covering 95 facilities found that 67% of emergency facilities were located in the least populated districts (33). However, this study also found that only 6% of basic health facilities were equipped to perform all EmONC signal functions, and only 3% of emergency facilities could perform all aspects of CEmONC. Moreover, 39% of maternal and neonatal mortality cases occurred at facilities incapable of providing basic emergency services (33).

## 3 Human resources

Human resources play a pivotal role in all aspects of EmONC services, significantly influencing its quality, particularly for complications requiring clinical procedures (34). In Ghana, highly skilled health workers tend to be concentrated in urban areas and work predominantly in public institutions, leaving rural and underserved regions with less skilled personnel (35).

Similar to other countries in Africa, Ghana has a pressing shortage of healthcare professionals (36). A study of the types and distributions of healthcare professionals found that 74% of CHPS zones failed to meet the requirement of having one midwife, although 91% of health centres did have at least one midwife (35). Further, only 29% of primary hospitals have an obstetrician-gynaecologist (35).

Greater Accra region boasts the highest ratios of obstetricians/gynaecologists (7.2), general practitioners (27.2), and midwives (62.6) per 200,000 people. In contrast, the Northern region has the lowest numbers, with only 0.4 obstetricians/gynaecologists, 3.2 general practitioners, and 25.0 midwives per 200,000 population. Most health centres, clinics, maternity homes, and CHPS zones typically have just one midwife or none (19).

For instance, data indicates that 42% of doctors are in Greater Accra alone, and up to 81% are concentrated in just five regions—Greater Accra, Ashanti, Central, Northern, and Volta (37). Teaching hospitals in these regions collectively house over 60% of all doctors in Ghana. This is largely due to the perception that urban areas offer better working conditions, including career development opportunities, lighter workloads, better clinical infrastructure, enhanced social life, improved accommodation, and higher income (38, 39). This uneven distribution results in unmet health needs in rural and remote areas, leading to lower life expectancy and higher rates of preventable maternal and under-5 mortality (37). Several studies in Ghana have reported the impact of unequal distribution of human resources of EmONC service (20, 31, 40, 41).

The delivery of EmONC depends on having personnel available and ensuring they are trained to perform the needed services (24). A systematic review focusing on the human resources aspect of EmONC revealed that staff shortages pose a significant barrier to delivering high-quality EmONC services (34). The GHFA report indicated that 71% of health facilities offered delivery services, with 67% providing 24/7 delivery care by a skilled provider (25). Additionally, skill deficiencies in essential healthcare professionals further exacerbate the challenge of delivering quality EmONC (20, 31). Typically, BemONC training is delivered as a 10-day course where healthcare professionals who provide direct maternal and neonatal care gather at a site away from their facility and receive both didactic and hands-on practice. This intensive experience, although effective, can be expensive and time-consuming and does not ensure that all personnel at a facility are trained in BEmONC (42).

Lastly, barriers to effective management of emergency referrals include inadequate healthcare personnel to manage patient management during transit, someone to accompany more stable patients, and provision of sufficient patient information to the receiving facility (41). These challenges were also highlighted by Afari et al. (2014), who emphasised that the scarcity of healthcare professionals results in limited clinical expertise and management, compromised quality of care, and inadequate supervision and interventions.

## 4 Supply chain logistics and equipment

EmONC's effectiveness heavily depends on the availability of supply chain logistics and equipment. These resources are crucial for providing timely and appropriate care to women and newborns in critical situations. Without an adequate supply of medications and equipment, health facilities may struggle to perform essential procedures and interventions, leading to increased risks of maternal and newborn morbidity and mortality. Additional factors are essential for the medications on hand to be effective. For example, effective oxytocin requires ensuring that the initial packing uses an adequate amount of the active ingredient and that the medication is stored at 2–8°C (43). A recent survey in Ghana found that 90% of health clinics and hospitals had a refrigerator to store oxytocin but only 40% of CHPS zones doing deliveries did have refrigerator (44). An older study analysed samples of two drugs for the prevention and treatment of postpartum haemorrhage and found the median percent of active ingredients were only 64% and 50% for oxytocin and ergometrine, respectively (45). Regarding the supply of logistics and equipment, the national assessment on EmONC reported that all teaching and regional hospitals are well-equipped, whereas only 74% of district hospitals, health centres, and CHPS zones have adequate supplies (19).

Recently, GHFA (25) found that the availability of essential life-saving commodities such as oxytocin injection, magnesium sulfate injection, and misoprostol tablets is high in hospitals, with coverage exceeding 96%. However, availability is significantly lower at health centres and CHPS compounds. Additionally, only 44% of health centres and 14% of CHPS have newborn resuscitation equipment. These commodities and equipment are expected to be available only in facilities staffed with midwives (25). Moreover, among facilities providing CEmONC services, only 63% of regional hospitals and 41% of district hospitals had a functional anaesthesia machine (25). A recent study among postpartum women in Ghana found deficiencies in medication and equipment, further compromising the delivery of high-quality care (20). According to a study by Daniels & Abuosi (2020), healthcare professionals also encountered obstacles to the referral process, including underutilisation of ambulance services and inadequate availability of beds, medications, and blood.

Emergency Transport System (ETS) serves as a critical link between healthcare facilities and families of women in need of urgent maternal transport. Community involvement is fundamental to improving emergency transport services. National data from Ghana in 2011 indicate that only 33% of health facilities rely on the national ambulance system for emergency referrals. In contrast, 51% use private transport options such as taxis or buses, while 46% expect clients to arrange their own transport. Among maternity units, 70% depend on private transport, whereas 62% of CHPS compounds assume clients will organise their own transportation (19). However, according to the 2022–2023 Ghana Harmonised Health Facilities Assessment Report, only 32% of health facilities nationwide have access to an emergency transport system. Access is particularly limited at the primary care level, with just 18% of CHPS compounds, 29% of health centres, 39% of polyclinics, and 40% of clinics and maternity homes equipped with such systems (25).

According to Oguntude et al. (46), community members perceive the ETS as reliable and responsive to women's emergency needs. Sunguya et al. (47) highlight that collaboration with local taxi drivers supports the timely referral of pregnant women, while the repair and upgrading of ambulances and coordination with receiving facilities further enhance the emergency response. A systematic review by Wilson et al. (48) identifies several potential solutions to maternal transport challenges, including motorcycle ambulance programmes, partnerships with taxi services, community education efforts, financial subsidies, and regular maintenance of vehicles.

## 5 Recommendations

There are still gaps in Ghana meeting national and global EmONC guidelines, so solutions are needed to address these disparities. A two-pronged approach is recommended. One recommendation focuses on upgrading existing facilities and recruiting and retaining healthcare professionals in rural and underserved areas. The second calls for an increase in the capability of delivery of EmONC by improving training efficiency and focusing on facilities missing only 1–2 signal functions.

### 5.1 Improving the provision of health care in rural and underserved areas

Through the Ministry of Health, the government must implement measures to improve underserved areas, including CHPS zones and a majority of the facilities in the Northern Region. Supply issues to CHPS zones should be a focus of efforts, so these facilities can meet the primary care needs of the community. Strategies for maintaining adequate electricity for the limited medication supply requiring refrigeration and supply chain issues need to be developed. Evidence shows that well-equipped health facilities assist with healthcare worker retention and satisfaction (49).

Policymakers need to develop effective deployment strategies to facilitate the movement of personnel to these high-need areas and ensure an equitable distribution of healthcare professionals. Experiments in other African countries have shown that higher wages and offering continuing education and career development opportunities are the most effective incentives (50). Healthcare workers, especially new graduates, want to be supported by a manager or mentor (51). Additionally, subsidised housing has also shown to be helpful in recruitment and retention. Ghana may want to develop its discrete-choice experiments in developing a tailored incentive package for recruiting and retaining healthcare workers in rural areas (52). In the long term, Ghana should consider a rural pipeline program that identifies students interested in healthcare in rural communities and provides tuition assistance for education (49). Additionally, programs should examine their curriculum to ensure that rural health subjects are taught and that students receive some training in rural health environments (49). When rural healthcare is part of training, retention and rural outcomes are improved as students often return to their communities where they have an intimate understanding of the social and cultural aspects of the community and its intersection with health (53).

Although Ghana's maternal health services are integrated into the NHIS, substantial gaps in human resources and logistical capacity hinder effective service delivery (25). To this end, adopting the cost-benefit analysis of priority health system strengthening interventions proposed by Asuning et al. (54) offers a strategic pathway for maximising health returns through targeted, efficient investments aligned with national health financing goals. The analysis found that over 15 years (2018–2032), addressing the national gap in EmONC facilities in Ghana is projected to cost approximately GH¢ 835 million, discounted at 8% (54). This cost estimate encompasses investments in staff capacity, logistical support, and minor extensions or renovations of existing infrastructure. In contrast, the projected economic benefits, primarily from averting neonatal deaths and reducing morbidity through nationwide EmONC scale-up, are estimated at GH¢ 5.8 billion (54).

### 5.2 Increase the capability of delivering EmONC

The traditional 10-day course for teaching EmONC should be replaced with an evidenced-based lower, intensity but high-frequency method. A cluster-randomized trial conducted from March 2014 to February 2017 across 40 public and mission hospitals in three regions of Ghana found this approach resulted in a sustained decrease in facility-based newborn mortality and intrapartum stillbirths (42). This approach provides two 2-day didactic and simulation sessions for all persons involved with birth at a facility. These 2-day sessions are a month apart and between sessions, participants receive SMS text messages with reminders and short quizzes as well as mentoring by an on-site person designated as an EmONC mentor. The SMS messaging continues for up to 10 months after the last 2-day session. For facilities with a small number of workers, an outside EmONC mentor could be identified who could be available and meet regularly by SMS messaging or telephone. This system would allow for a less costly and more efficient method of training a greater number of healthcare workers in EmONC and maintaining their skills.

A geospatial study by Bosomprah and colleagues showed the most efficient strategy for having the greatest benefit in decreasing the number of poor maternal and neonatal outcomes would be to prioritise improving facilities that were missing only 1 or 2 signal functions first (30). Unfortunately, their study mapped facilities that were surveyed in 2010. For this to be an effective strategy, a new survey of birthing facilities would be needed to update the information to proceed. There are a few studies that have conducted more limited surveys of EmONC services that could be used as a starting point in a prioritisation list (30–33).

## 6 Limitation

This critical analysis is constrained by limited data availability, relying primarily on findings from the National assessment for EmONC in Ghana conducted in 2011 (19), which may not accurately reflect the current state of EmONC services in Ghana. However, more recent data from the 2022–2023 GHFA (25) report were incorporated to supplement and update the earlier information.

## 7 Conclusions

Improving maternal and child health remains a top priority for the Ghanaian Ministry of Health. Despite substantial progress, a substantial gap exists in facilities providing functional EmONC services. Ghana continues to face significant challenges concerning infrastructure, skilled personnel, equipment and supplies to support EmONC in decreasing maternal and newborn morbidity and mortality.

Addressing these challenges requires comprehensive efforts to improve infrastructure, human resources, and supply chain logistics support. Upgrading facilities that lack just one or two signal functions will enable Ghana to approach the global criteria for the accessibility of EmONC services (30) and will prevent an estimated 6,666 neonatal deaths and 28,486 annual neonatal deaths over a 15-year implementation period (54).

## Data Availability

The original contributions presented in the study are included in the article/Supplementary Material, further inquiries can be directed to the corresponding author.
